# Hibernating Little Brown Myotis (*Myotis lucifugus*) Show Variable Immunological Responses to White-Nose Syndrome

**DOI:** 10.1371/journal.pone.0058976

**Published:** 2013-03-20

**Authors:** Marianne S. Moore, Jonathan D. Reichard, Timothy D. Murtha, Morgan L. Nabhan, Rachel E. Pian, Jennifer S. Ferreira, Thomas H. Kunz

**Affiliations:** Department of Biology, Center for Ecology and Conservation Biology, Boston University, Boston, Massachusetts, United States of America; The University of Wollongong, Australia

## Abstract

White-nose syndrome (WNS) is an emerging infectious disease devastating hibernating North American bat populations that is caused by the psychrophilic fungus *Geomyces destructans*. Previous histopathological analysis demonstrated little evidence of inflammatory responses in infected bats, however few studies have compared other aspects of immune function between WNS-affected and unaffected bats. We collected bats from confirmed WNS-affected and unaffected sites during the winter of 2008–2009 and compared estimates of their circulating levels of total leukocytes, total immunoglobulins, cytokines and total antioxidants. Bats from affected and unaffected sites did not differ in their total circulating immunoglobulin levels, but significantly higher leukocyte counts were observed in bats from affected sites and particularly in affected bats with elevated body temperatures (above 20°C). Bats from WNS-affected sites exhibited significantly lower antioxidant activity and levels of interleukin-4 (IL-4), a cytokine that induces T cell differentiation. Within affected sites only, bats exhibiting visible fungal infections had significantly lower antioxidant activity and levels of IL-4 compared to bats without visible fungal infections. Overall, bats hibernating in WNS-affected sites showed immunological changes that may be evident of attempted defense against *G. destructans*. Observed changes, specifically elevated circulating leukocytes, may also be related to the documented changes in thermoregulatory behaviors of affected bats (i.e. increased frequencies in arousal from torpor). Alterations in immune function may reflect expensive energetic costs associated with these processes and intrinsic qualities of the immunocapability of hibernating bats to clear fungal infections. Additionally, lowered antioxidant activity indicates a possible imbalance in the pro- versus antioxidant system, may reflect oxidative tissue damage, and should be investigated as a contributor to WNS-associated morbidity and mortality.

## Introduction

Successful resistance against pathogen invasion involves the coordinated elevation of multiple innate and adaptive immune mechanisms, some of which may be particular to the type of invading organism. Although there is no single method to assess the overall elevation of immune response in reaction to pathogen invasion, quantifying the relative concentrations of immune function components (e.g. circulating leukocytes, immunoglobulin and cytokines) may provide evidence of which mechanisms have been activated, especially when compared to corresponding levels observed in healthy individuals of the same species. This process is complicated when the ecology of a newly emerging infectious disease is difficult to study (e.g. host and pathogen are adapted to relatively extreme conditions, host is widespread and free-ranging) and further challenged when little is known about the immune systems of affected species. These difficulties may result in slow elucidation of the role of immune responses in the pathogenesis of a particular disease because methods must be modified and validated for testing affected species and baseline data must be collected at the same time disease processes are being investigated.

Unfortunately, a devastating disease emerging in North American hibernating bat species known as white-nose syndrome (WNS) [1] has caused the deaths of an estimated 5.7 to 6.7 million bats [2] and is threatening at least one species with regional extinction [3], [4]. Characteristics associated with the syndrome include low fat reserves [1], [5], [6] to emaciation [7], increased frequency in arousal from torpor [7], [8] and atypical behavior such as roosting or flying outside of hibernacula in mid winter (Alan Hicks, pers. comm.). Additionally, severe wing damage is observed primarily early in the active season [9], [10]. However, the diagnostic and most notable symptom of WNS is a cutaneous fungal infection that manifests itself as a white, filamentous growth on the muzzle, and powdery growth on the surfaces of ears, wings and tail membranes of hibernating bats [1], [6]. The agent causing this characteristic fungal infection and the cause of the syndrome is a recently identified psychrophilic fungus named *Geomyces destructans* [11], [12], which is adapted to the cold and humid conditions typical of hibernacula (i.e. site of hibernation) and proliferates optimally at a temperature range of 5–10°C [1]. *G. destructans* causes cupping erosions and ulceration of epidermal tissues, destruction of underlying connective tissue, and invasion of sebaceous and apocrine glands, as well as hair follicles [6]. However, according to an early study, bats with extensive fungal invasion collected during the hibernation period demonstrated scant presence of inflammation in the skin [6]. In contrast, bats exhibiting wing damage and sampled outside of affected hibernacula in early spring showed evidence of cutaneous cellular immune responses with suppurative dermatitis and serocellular crusts containing fungal hyphae [6]. To date, presence of *G. destructans* has been confirmed on nine species of bats in the family Vespertilionidae (*Myotis lucifugus*, *M. septentrionalis*, *M. leibii*, *M. sodalis*, *M. velifer*, *M. griscecens*, *M. austroriparius*, *Eptesicus fuscus*, *Perimyotis subflavus*; [1], [11], [13]), all of which hibernate for a significant fraction of the year [14]. *G. destructans* has also been observed or isolated from multiple bat species in Europe [15], [16], [17], [18] and recent studies confirmed several European bats with lesions characteristic of WNS [19]. Notably, no major mortality events have been observed in Europe and Warneke et al. suggest *G. destructans* may have been recently introduced to North America from this region [7]. Although some aspects of bat immune function have been described [20], [21], [22], [23], there is a general lack of knowledge regarding their responses particularly to pathogen invasion [24], [25], [26], [27], [28], [29]. Additionally, virtually nothing is known about how bat immune responses vary with season and the use of daily and/or seasonal torpor.

Although little is known about the mechanisms involved in skin immune responses in bats [30], there is a probable set of responses activating against this fungal pathogen if bats respond to invading fungi using similar mechanisms as other taxa. Specifically regarding invasion through the skin, these mechanisms should include phagocytosis by resident and recruited innate immune cells (i.e. macrophages, neutrophils), the respiratory burst, edema, vascular reaction and an increase in acute-phase proteins [31]. Activation of complement proteins may also occur in the stratum corneum [32] and dendritic cells and mast cells may be activated through Toll-like receptors [33]. Effector functions mediated by T lymphocytes and the development of immunological memory specific to *G. destructans* would almost certainly be essential for effective resistance [34]. Additionally, antibody-dependent cellular cytotoxicity might also play a role in clearance of *G. destructans* and further establishment of immunological memory [35].

Unfortunately, given the paucity of information regarding bat immune responses against fungal pathogens [6], [36], [37], we cannot predict which of the outlined responses typically involved in other taxa are likely to be observed in WNS-affected bats. Furthermore, regardless of which mechanisms are involved in responses against fungal pathogens in bats, many aspects of their immune function are probably reduced during hibernation [38], [39], [40], [41] and should therefore be less effective at clearing infection during this time. In part, this latter characteristic may account for the extreme and widespread mortality observed in this newly emerging infectious disease.

To help understand the pathogenesis of WNS, we evaluated variability in immune function among bats hibernating in WNS-affected and unaffected sites. Previously, we reported on the bactericidal and fungicidal ability of blood collected from little brown myotis (*Myotis lucifugus*) hibernating in sites affected by WNS compared with bats collected from unaffected sites [36]. We found that little brown myotis hibernating in WNS-affected sites experience significant changes in complement protein activity (elevated against *Escherichia coli* and *Staphylococcus aureus*, but reduced against *Candida albicans*). We observed that these alterations are also related to hibernation stage (i.e. early, mid-, or late winter) and body condition. In the current study, we continued with a similar line of research using multiple measures of immune function to test the following hypotheses: (1) that WNS-affected bats are elevating immune parameters presumably in response to *G. destructans*, (2) that immune responses are positively related to body temperature, or state of torpor versus arousal, and (3) that immune responses are positively related to body condition, or available energy stores. Testing these hypotheses are critical for understanding the pathogenesis of WNS since attempted but unsuccessful immune responses against *G. destructans* may contribute to mortality in itself and lead to depletion of critical energy stores that further reduce the health status of WNS-affected bats.

## Methods

We used blood-based methods that could be applied to samples stored frozen because the logistics of collecting bats both within and outside the affected region precluded our ability to test fresh tissues. We also focused on the most efficient use of blood samples, since even when using terminal sampling methods only very small volumes (∼150 µL whole blood) can be collected from animals of this size (6–9 g), especially while they are torpid. Using each of these methods, we tested blood from the same individuals used in our previous study and compared little brown myotis captured from WNS-affected and unaffected sites throughout the 2008–2009 hibernation period.

### Ethics Statement

Capture, handling and sample collection protocols for this study were reviewed and approved by the Boston University IACUC (protocol #08–022) and the US Fish and Wildlife Service Disinfection Protocol for Bat Studies was used for all collections. Authorized state biologists with whom we worked directly permitted all sample collections for our work in New Jersey, New York, and Pennsylvania. Protocols were permitted in Massachusetts (Permit #167.08 SCM), Michigan (Permit #SC620) and Vermont (10 VSA Section 5408: Thomas H. Kunz, 2008–09). Bats were collected by hand from roost substrates, individually placed in cloth bags, and sacrificed by decapitation.

### Collection and Sampling Methods

Adult female little brown myotis were collected from the following affected sites during the winter of 2008–2009: William’s Hole Six Mine, Ulster County, New York State on December 17, 2008 (n = 18); Aeolus Cave, Bennington County, Vermont on 18 November, 2008, 31 January, 2009, and 27 March, 2009 (n = 58); Chester Mine, Hampden County, Massachusetts on 20 November, 2008, 2 February, 2009 (n = 37); Hibernia Mine, Morris County, New Jersey on 13 January, 2009, 16 March, 2009 (n = 38). Adult female little brown myotis were collected from the unaffected CS&M Mine, Lawrence County, Pennsylvania on 21 January 2009, 17 March 2009 (n = 36) and Jones Adit/Vulcan Mines, Dickinson County, Michigan on 25 January 2009 (n = 19). For the purposes of this study we considered November and December as early hibernation, January and February as mid-hibernation, and March as late hibernation.

On each sampling date, 18 adult female little brown myotis were collected, six each corresponding to the following body temperature categories: 1) torpor: <10°C, 2) rewarming phase: 10–20°C and 3), euthermia: >20°C. Arousal from torpor to achieve these T_b_’s was assisted using an electric heating pad and insulated bag. Rectal T_b_ was measured using a small thermocouple (Omega HH82A, Stamford, CT) inserted before and after individuals were sacrificed by decapitation. Once a bat reached the desired temperature it was sacrificed immediately, except in the case of bats sampled near 37°C, which were held for no longer than 30 minutes before sacrifice. Because hibernating bats naturally undergo passive rewarming from arousing or euthermic bats roosting adjacent to them [42] it is unlikely this manipulation introduced an additional stressor. General life history data were collected, including body mass (g) and length of forearm (mm) and all bats were visually inspected for fungal infections and wing damage [9]. Individuals that exhibited obvious signs of fungal growth were defined as symptomatic; individuals that did not exhibit such signs were considered asymptomatic for the purposes of our comparisons. Blood was collected into 70 mm heparinized capillary tubes and centrifuged for 3 minutes to separate plasma from formed elements. Hematocrit was estimated using a card style hematocrit reader. Separately, plasma and red blood cell fractions were frozen on dry ice or in a dry nitrogen dewer during field collection and transportation, then stored at −80°C until assays were performed.

### Blood Smear Preparation and Analysis

We analyzed blood smears to quantify total circulating leukocytes and to detect potential changes in concentrations of circulating leukocytes as they migrate from other tissues through the periphery to the site of infection. A small sample (∼5 µL) of fresh whole blood was used to prepare a single blood smear for each individual. Our initial attempts to prepare blood smears directly after sacrifice failed due to the extreme humidity in caves and mines. We therefore held blood samples in an ice bath until we exited hibernacula and reached drier conditions, which delayed preparation of smears for no longer than 8 hours after sacrifice at some sites. After preparation, blood smears were air-dried for 30 minutes and fixed in 100% methanol. A methylene blue – eosin based stain kit (Hema-3 Stain, ThermoFisher Scientific 22-122-911) comparable to the Wright-Giemsa method was used to differentially stain leukocytes and, after another period of drying, a coverglass was permanently affixed to the slide using Permount (ThermoFisher Scientific SP15). Final cell counts were performed blind to the condition of the bat by MSM. We initially visualized slides at 1000× (Boreal Inc. #DMB1–223) with the intention of performing differential leukocyte counts, however, the majority of leukocytes present on many slides were unidentifiable to the level of cell type. Possibly because of the difficulty in working with blood from hibernating bats, the delay in blood smear preparation, the extremely low leukocyte population, or the health status of certain individuals, differential counts were not possible. We therefore performed total leukocyte counts at 400x. Mean number of leukocytes per high dry field was determined using the total count of leukocytes in 10 fields, or the number of fields that was necessary to examine until a single leukocyte was observed (e.g. some samples required visualizing between 40 and 60 fields, one required visualizing 95 fields before a single leukocyte was observed). To the best of our knowledge there is no defined correction factor to convert average number of leukocytes per field to leukocytes per µL in bats. We therefore express total leukocyte counts as mean number cells per field.

### Total Circulating Immunoglobulins

We developed a sandwich-based enzyme linked immunosorbant assay (ELISA) to estimate total circulating immunoglobulins of all isotypes and to detect potential activation of B lymphocytes and generation of antibodies presumably against *G. destructans*. The assay was developed using affinity purified bat IgG-heavy and light chain antibody (capture antibody; product # A140-118A) and affinity purified horseradish peroxidase (HRP) conjugated bat IgG-heavy and light chain antibody (primary antibody; product # A140-118P), both produced by Bethyl Laboratories Inc., Montgomery, Texas. Ninety-six well plates were used to minimize volumes, particularly that of plasma, in the assay. Wells were coated with 100 µL capture antibody solution using bat IgG antibody diluted 1∶100 in coating buffer (Bethyl Laboratories Inc. # E105), then incubated at room temperature for 60 minutes, aspirated and washed 5 times with wash solution (Bethyl Laboratories Inc. # E106) using a bench-top aspirator (IBS Integra Biosciences, Vacusafe Comfort Aspirator). Wells were blocked using 200 µL postcoat/blocking buffer (Bethyl Laboratories Inc. # E104), incubated for 30 minutes at room temperature, aspirated and washed 5 times. Plasma samples were diluted 1∶1000 using 1 µL plasma in 999 µL sample diluent (ELISA blocking buffer plus 0.05% Tween 20, Bethyl Laboratories Inc. # E108). A little brown myotis plasma pool was used for quality control, aliquots of which were also diluted 1∶1000. In duplicate, 100 µL of diluted samples or quality controls were dispensed into wells, microtiter plate was incubated at room temperature for 60 minutes, and wells were again aspirated and washed 5 times. Primary antibody dilution was prepared using HRP conjugated bat Ig antibody diluted 1∶5,000 in conjugate diluent (Bethyl Laboratories Inc. # E108). 100 µL of primary antibody dilution was dispensed in each well, and the plate was incubated at room temperature for 60 minutes, aspirated and washed 5 times. Enzyme substrate solution was prepared using a 1∶1 TMB (3, 3′, 5, 5′ tetramethyl benzidine) and peroxidase substrate (Bethyl Laboratories Inc. #E102). 100 µL of enzyme substrate solution was dispensed into each well, and the plate was incubated at room temperature in the dark for 25 minutes, at which point the solution turned blue. To stop the reaction, 100 µL 2 M H_2_SO_4_ was dispensed into each well, the plate was tapped gently to mix and the solution turned yellow. The underside of the plate was wiped with a lint-free cloth and absorbance was measured at 450 nm using a microtiter plate reader (Bio-Rad Laboratories Inc., Model # 680). To standardize results from multiple assays, mean absorbances were multiplied by their dilution factor and a coefficient of variation, which was calculated for each assay using the interassay variation in quality control samples [43]. Percent differences between sample duplicates were all within 10%, except four samples that had percent differences greater than 10% but less than 15%. Interassay variation was 13.7%.

### Total Antioxidant Power

We measured total antioxidant activity to estimate the maintenance of antioxidants in the system. We conducted this analysis to test for the capacity of this defense system to protect the self from the destructive effects of free-radical production. This destruction could either happen during the respiratory burst or during the massive increases in mitochondrial respiration that occur during the rewarming phase, which notably occurs more frequently in WNS-affected bats [7], [8]. A colorimetric assay kit (Oxford Biomedical Research #TA02d) based on the ability of antioxidants to convert Copper (II) to Copper (I) was used to estimate total antioxidant power (TAP) or the collective activity of all antioxidants in the red blood cell fraction of blood samples. For each sample, erythrocyte volume was measured to the nearest microliter using a standard metric ruler, equating each millimeter in capillary tube length to one microliter in erythrocyte volume. Erythrocytes were then expelled from their capillary tubes using a bulb assembly, and lysed by diluting each sample 1∶10 in ice-cold deionized water. Because the chromogenic reagent used in the assay kit exhibits an absorption maximum at 450 nm [44] and hemoglobin absorbs wavelengths between 340 and 600 nm [45], hemoglobin was precipitated by adding 0.25 volumes 98% ethanol and 0.15 volumes chloroform to each diluted sample. Following the addition of ethanol and chloroform, samples were vortexed for approximately 1 min and then centrifuged at 10,000 rpm for 10 min at 4°C. The clear supernatant was collected and frozen at −80°C for no more than 1 week, then assayed following the kit protocol without modification. Absorbance was measured using a microtiter plate reader (Bio-Rad Laboratories Inc., Model # 680). Net absorbance values were calculated by subtracting the mean of each sample or standard’s reference measurements from the mean of each sample or standard’s corresponding final absorbance measurements. TAP values were initially expressed in terms of µM uric acid equivalents using each sample’s net absorbance divided by the value for the slope of the uric acid standard curve. Values were further converted into µM copper reducing equivalents (CREs) by multiplying each uric acid equivalent by a given constant, 2189 µM.

### Circulating Levels of Various Cytokines

A bead-based immunofluorescence assay developed for detecting mouse analytes (Luminex Inc., Austin, TX) and using multiplex cytokine kits and reagents (R&D Systems, Minneapolis, MN) was used to estimate plasma levels of the following cytokines: IFN-γ, IL-1β, IL-2, IL-4, IL-6, IL-10, IL-12p70, IL-13, and TNF-α. These cytokines were chosen to detect aspects of the fever, acute phase, inflammatory and anti-inflammatory responses (IL-1β, IL-6, TNF-α, and IL-10) as well as T cell subset responses (T_H_1: IFN-γ, IL-2, IL-12p70; T_H_2: IL-4, IL-6, IL-10 and IL-13). This method employs a sandwich immunoassay-based protein array system containing dyed microspheres conjugated with a monoclonal antibody specific for a target protein. Plasma samples were thawed, diluted to 1∶4, and assayed in duplicate. Following the kit protocol, antibody-coupled beads were first incubated with the plasma sample then with biotinylated detection antibody before finally being incubated with streptavidin-phycoerythrin. A broad sensitivity range of standards (R&D Systems), ranging between 7.09 and 50,300 pg/mL depending on the cytokine were used to increase sensitivity. Captured bead-complexes for both samples and standards were detected using a Luminex 200 array reader at the Harvard Medical School Department of Genetics Biopolymers Facility. This instrument uses fluorescent bead-based technology with a flow-based dual laser detector and real-time digital signal processing that allows for the simultaneous detection of multiple cytokines in a single sample.

### Statistical Analysis

Total immunoglobulin levels (total Ig) were normally distributed (Shapiro-Wilks test p = 0.79). We used a parametric mixed model ANOVA/ANCOVA for this dataset and tested the following independent variables for significance: site, perpetual date, hibernation stage (i.e. early, mid, late hibernation) for samples collected from the affected Aeolus Cave only, T_b_, hematrocrit, body mass index (BMI; log body mass (g)/log length of forearm (mm)), site type categorized as WNS-affected versus unaffected, and individuals categorized as symptomatic versus asymptomatic (from affected sites only and based on the presence or absence of visible cutaneous fungal infections characteristic of WNS). We tested for interactions and used a nested design to test for differences in total Ig between affected and unaffected sites beyond individual site differences. We used Type IV sums of squares to account for uneven sample sizes between groups and an unbalanced design with missing treatments (e.g. no unaffected bats were sampled during early hibernation). Comparisons of interest were extracted using simple contrasts and each contrast was examined for significance. We used Levene’s test of unequal variance and Brown-Forsythe and Welch’s tests to assess significance when Levene’s p<0.05. To further determine differences and relationships between immune response and variables included in the reduced model, we used Tukey’s post hoc analysis.

Total leukocyte counts (Shapiro-Wilks test p<0.001), TAP levels (Shapiro-Wilks test p<0.05) and cytokine levels (IL-4; Shapiro-Wilks test p<0.001) were not normally distributed. Because transformations did not improve normality for these measures, statistical analysis was conducted using non-parametric tests including the Kruskal Wallis test, the Mann-Whitney U test, and the Spearman’s Rho test. Two extreme values were excluded from TAP analysis. SPSS (16.0.2, 2008) was used for all analyses with α = 0.05.

## Results

Test statistics for all analyses are indicated in Table 1. For greater detail, including distribution of samples across groups, see Table S1.

**Table 1:** Statistics for all outcome variables used in this study; see Table S1 for means and distribution of samples across groups used in comparisons.

Variable	Circulating Leukocytes (WBC)	Total Immunoglobulin (Tig)	Total Antioxidant Power (TAP)	IL-4
Site Type – all samples	§ ***U = 1336; p = 0.008; N = 134***	€F = 0.984; p = 0.33; n = 66	***U = 1877; p<0.001; n = 191***	***U = 285; p = 0.035; n = 73***
Site Location – all samples	‡χ2 = 9.706; p = 0.08; n = 134	***F = 8.248; p<0.001; n = 148***	***χ2 = 23.06; p<0.001; n = 191***	χ2 = 9.126; p = 0.058; n = 73
Hibernation Stage – affected Aeolus Cave only	χ2 = 1.541; p = 0.46; n = 94	***F = 4.962; p<0.001; n = 54***	χ2 = 4.414; p = 0.11; n = 56	χ2 = 3.30; p = 0.22; n = 15
Body Temperature (measurment) – all samples	◊ρ = 0.13; p = 0.13; n = 134	F* = *0.03; p = 0.86; n = 148	ρ = 0.074; p = 0.31; n = 191	ρ = -0.19; p = 0.12; n = 73
affected sites	ρ = 0.18; p = 0.08; n = 94	F* = *3.33; p = 0.07; n = 114	ρ = 0.07; p = 0.41; n = 147	ρ = -0.25; p = 0.06; n = 59
unaffected sites	ρ = -0.18; 0.91; n = 40	***F = 4.962; p = 0.034; n = 34***	ρ = 0.03; p = 0.86; n = 44	ρ = -0.14; p = 0.63; n = 14
Body Temperature (categorical) – allsamples	χ2 = 4.708; p = 0.09; n = 134	F* = *1.09; p−0.34; n = 148	χ2 = 4.708; p = 0.09; n = 191	χ2 = 5.17; p = 0.08; n = 73
affected sites	***χ2 = 3.586; p = 0.022; n = 94***	F* = *1.78; p = 0.17; n = 114	***χ2 = 3.586; p = 0.022; n = 147***	χ2 = 4.872; p = 0.09; n = 59
unaffected sites	χ2 = 3.586; p = 0.17; n = 40	***F = 3.33; p = 0.049; n = 34***	χ2 = 3.586; p = 0.17; n = 44	χ2 = 0.386; p = 0.83; n = 14
Hematocrit – all samples	ρ = 0.039; p = 0.66; n = 196	F* = *1.12; p = 0.29; n = 148	ρ = 0.039; p = 0.66; n = 195	ρ = −0.005; p = 0.97; n = 73
affected sites	ρ = 0.031; p = 0.77; n = 93	F* = *0.68; p = 0.41; n = 114	ρ = 0.031; p = 0.77; n = 93	ρ = 0.031; p = 0.77; n = 93
unaffected sites	ρ = −0.09; p = 0.58; n = 68	F* = *0.548; p = 0.47; n = 34	ρ = −0.09; p = 0.58; n = 70	ρ = −0.09; p = 0.58; n = 70
Visible Symptoms – mid-hibernation only	U = 1013; p = 0.75; n = 92	F* = *1.262; p = 0.26; n = 117	***U = 1899; p = 0.009; n = 144***	***U = 287; p = 0.048; n = 59***
Body Mass Index – all samples	ρ = −0.08; p = 0.36; n = 133	F* = *0.07; p = 0.8; n = 148	***ρ = 0.16; p = 0.035; n = 179***	ρ = 0.44; p = 0.12; n = 73
affected sites	ρ = 0.09; p = 0.4; n = 92	F* = *1.75; p = 0.19; n = 114	ρ = 0.11; p = 0.21; n = 141	ρ = 0.06; p = 0.69; n = 57
unaffected sites	ρ = −0.11; p = 0.5; n = 38	F* = *1.713; p = 0.2; n = 34	ρ = 0.08; p = 0.63; n = 43	ρ = 0.44; p = 0.12; n = 14

Key:

§ Mann-Whitney U;

‡ Kruskal-Wallis;

◊ Spearman correlation;

€ Mixed model ANOVA/ANCOVA.

### Total Circulating Leukocytes

**Figure 1:**
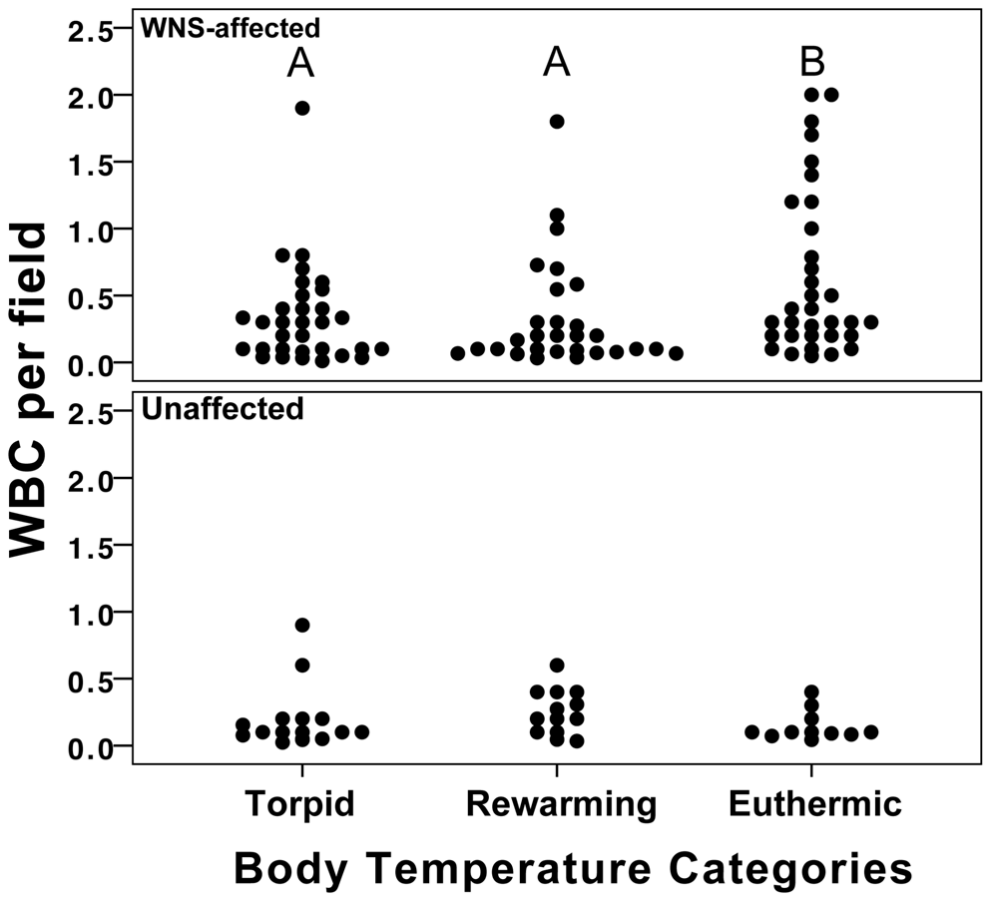
Total circulating leukocytes in WNS-affected and unaffected little brown myotis. Total circulating leukocytes (WBC) were estimated in little brown myotis collected from WNS-affected and unaffected sites during the winter of 2008–2009. Bats were sampled at body temperatures (T_b_) indicative of torpor, the rewarming phase and euthermia. WBC was significantly related to temperature category in affected bats (Kruskal Wallis χ^2^ = 7.61, p = 0.022), but not in bats from unaffected sites (Kruskal Wallis χ^2^ = 3.586, p = 0.17). In affected bats, WBC was highest in individuals with T_b_>20°C (torpid n = 32, rewarming n = 29, euthermic n = 33). Data points represent individuals and are spread for clarity. Groups labeled with different letters are significantly different from one another at the α = 0.05 level.

Total circulating leukocyte counts (WBC) were significantly higher in bats from affected sites compared with bats from unaffected sites. Within affected sites, WBC did not vary between symptomatic and asymptomatic bats. Across all samples, WBC did not vary depending on hibernation stage and also did not vary depending on hibernation stage within groups representing bats from affected or unaffected sites. Hematocrit was not significantly related to WBC across all samples or within groups representing bats collected from WNS-affected and unaffected sites respectively. T_b_ was marginally correlated with WBC in bats collected from affected sites, but was not correlated with WBC in bats from unaffected sites. To investigate this possible relationship further, we categorized T_b_ based on temperatures that were indicative of torpor, the rewarming phase, or euthermia. WBC was significantly related to temperature category specifically in affected bats, but not in bats from unaffected sites. In affected bats, WBC was highest in individuals with T_b_>20°C (**Figure 1**). WBC did not vary depending on BMI in bats from affected or unaffected sites. This lack of a relationship held true in each group throughout the entire hibernation period as well as within each hibernation stage (i.e. early, mid-, late).

### Total Immunoglobulins

The reduced model including site and hibernation stage significantly explained variation in total immunoglobulins (TIg; N = 154; F*_7, 154_* = 7.228; p<0.001; R^2^ = 0.26; adjusted R^2^ = 0.22). Whether a site was affected by WNS did not significantly predict TIg. Because we were able to collect samples throughout the hibernation period only from one site (Aeolus Cave, VT), we used data from this site to test variation between early, mid-, and late hibernation. TIg varied throughout the hibernation period (early: 1.836±0.209 corrected OD, n = 15; mid: 2.115±0.225 corrected OD, n = 13; late: 1.777±0.193 corrected OD, n = 16) and was highest during mid-hibernation as compared with early (p = 0.003) and late hibernation (p<0.001). We observed no difference in TIg between visibly symptomatic and asymptomatic bats within affected sites and no relationship between TIg and BMI, hematocrit, or T_b_.

### Total Antioxidant Power

Bats from WNS-affected sites exhibited significantly lower total antioxidant power (TAP) compared to bats from unaffected sites. Within affected sites, symptomatic bats had significantly lower TAP compared to asymptomatic bats. Across samples collected only from Aeolus Cave, TAP did not vary depending on hibernation stage. Across all samples, T_b_ was not correlated with TAP levels, nor did it correlate with TAP levels when we analyzed bats from affected and unaffected sites separately. BMI did not correlate with TAP levels in bats from affected or unaffected sites when all hibernation stages were analyzed together. However, BMI was significantly and negatively correlated with TAP specifically in bats from affected sites during mid-hibernation. TAP did not correlate with hematocrit.

### Circulating Cytokines

Of the nine cytokines that we attempted to detect using the multiplex cytokine array with mouse specific reagents, IL-4 was the only analyte that was detected with any consistency. We therefore focused on this cytokine alone in our analyses. Circulating levels of IL-4 were greater in bats collected from unaffected sites as compared with bats from affected sites. Within affected sites only, IL-4 levels were significantly lower in bats displaying visible fungal infections characteristic of the syndrome. IL-4 did not differ depending on hibernation stage in bats collected from Aeolus Cave. Across all samples, IL-4 did not differ depending on BMI, nor did it vary depending on BMI when we analyzed bats from affected and unaffected sites separately. Across all samples, IL-4 levels did not correlate with T_b_, but when we analyzed the data set using temperature categories there was a negative trend between circulating IL-4 and stage of torpor vs. arousal. We therefore looked at this potential relationship within bats from affected and unaffected sites separately and observed the negative trend in affected bats, but not in unaffected bats. Finally, we observed no relationship between IL-4 levels and hematocrit.

## Discussion

Our observations of increased WBC and decreased TAP suggest an inflammatory response is stimulated in some WNS-affected bats [46], [47], [48]. These results apparently contradict those of Meteyer et al. [6], where little inflammation was observed in the cutaneous tissues of infected bats sampled during hibernation. However, our results may reflect unsuccessful attempts to defend against *G. destructans*, attempts that result in a signal of increased immune activation in the blood (i.e. increased circulating leukocytes), but do not translate to similar observations downstream (i.e. in cutaneous tissues where these cells should be recruited). This putative futile attempt may be a critical mechanism underlying mortality, since it is likely that the observed immune activation would require the expenditure of limited energy reserves without the subsequent benefits of pathogen clearance. It is also possible that bats exhibiting immune activation were capable of resisting *G. destructans* and surviving the syndrome and would have exhibited recruitment of immune cells to the site of invasion had we sampled these tissues. Given that the 5-year average population decline of little brown myotis is 91% [49], only 9% of individuals of this species appear to be capable of surviving exposure (although it remains unconfirmed if remaining bats are in fact survivors and not immigrants to the surveyed populations). Still, immunologically resistant individuals would be difficult to detect without unrealistically large sample sizes, although it is possible that we captured some of these individuals. Lack of observed increases in TIg in affected bats more directly corroborates Meteyer et al.’s analysis of cutaneous tissue sections. If cells were successfully recruited to the site of invasion and subsequent mechanisms activated, we might have observed higher levels of TIg in affected bats. However, given the lack of cells present in previously sampled cutaneous tissues, it is probable that there is also a lack of T and B cell activation (at least during hibernation), which our results support. Our ability to interpret IL-4 results is limited, particularly since we do not know the normal baseline levels in this species or have results of other cytokines. However, our results do show alterations in this cytokine suggesting that T cell responses are potentially induced.

### Total Circulating Leukocytes

We estimated extremely low leukocyte population counts in hibernating little brown myotis across our sample set and observed significantly higher WBC in WNS-affected bats sampled at temperatures corresponding to euthermia (T_b_>20°C) compared with rewarming (T_b_<20°C) and torpor (T_b_<10°C). This change in circulating leukocytes did not correlate with hematocrit, suggesting it was not related to hydration status of the individuals sampled, nor was a similar pattern observed in samples from unaffected bats. We did not observe differences in WBC between visibly symptomatic and asymptomatic bats within affected sites. It seems that, if obvious signs of fungal infection were an indication of infection status, we would have observed some indication of this in cell counts. One possible explanation is that individuals without visible evidence of fungal infections may have groomed all external fungus from their skin during more recent arousals from torpor. Alternatively, these individuals may have been in the earlier stages of fungal infection before hyphae and conidia were manifested and before additional leukocytes could be produced or recruited.

We were unable to conduct differential leukocyte counts because of the extremely low white blood cell populations present on blood smears and because of other factors likely associated with hibernation physiology (e.g. viscosity of blood), or possibly suboptimal field conditions (e.g. extreme humidity). A similar difficulty was encountered by Krutzsch and Hughes [41] when they compared hematological values in active and torpid *Tadarida brasiliensis* and *Myotis velifer*. They were able to quantify total circulating leukocytes, but were unable to perform differential counts, and observed an overall decrease in circulating leukocytes in torpid bats compared with active bats. Bouma et al. [50] also observed significant decreases in leukocyte populations in hibernating European ground squirrels (*Spermophilus citellus*), which returned to euthermic summer levels 1.5 hours after arousal. In unaffected (i.e. control) bats, we did not observe elevated WBC associated with euthermia as would be expected. Our samples were collected within 30 minutes of animals reaching a T_b_ indicative of euthermia, which may have been too soon to detect a return to normothermic values. However, bats typically rewarm to euthermia more rapidly than other hibernators and may have arousal bouts under 90 minutes [51] making it difficult to predict changes in circulating leukocytes given the extent to which they differ from other taxa.

### Total Circulating Immunoglobulins

The role of antibody immunity in controlling invasive fungal infections has been difficult to determine in any taxon [52], [53]. Support for the protective aspect of antibodies has been equivocal, and a number of studies have produced contradictory results [54]. Nonetheless, there is a body of evidence showing that antibodies prevent the adherence of fungi to tissue surfaces, neutralize toxins produced by fungi, function as opsonins, and mediate antibody-dependent cellular cytotoxicity [53].

Our observations of total Ig (TIg) in little brown myotis show that bats hibernating in sites affected by WNS have TIg levels no different from bats hibernating in unaffected sites. This suggests that affected bats are capable of maintaining similar TIg levels throughout the hibernation period as compared with controls. Further research needs to be performed to determine whether or not little brown myotis are capable of producing anti*-G. destructans* antibodies. These studies should be conducted during the active season when there are fewer constraints on the immune system before being pursued during the hibernation period.

### Total Circulating Antioxidants

Upon detection of microbial intruders, phagocytes of the host’s innate immune system increase their consumption of molecular oxygen through the activity of NADPH oxidase. This oxidative burst results in the production of metabolites that propagate strongly anti-microbial free radicals such as various reactive oxygen and nitrogen species [48], [55]. To protect the self, antioxidants are produced by the host and neutralize the damaging effects of free-radical production during the inflammatory process [56], [57]. Tissue concentrations of these free-radical scavengers are known to vary depending on infection status [58], [59], [60] and disease state [61], [62], [63]. When the rate of free radical production exceeds the antioxidant capacity of an organism, this imbalance, known as oxidative stress, results in damage to DNA, proteins, and lipids [48], [64]. Another component of the anti−/pro-oxidant system as it may relate to WNS involves the extreme fluctuations in oxygen consumption of bats while they periodically transition from deep torpor to euthermia [65]. During arousals, the rapid rates of oxygen consumption in brown adipose tissue (BAT) promote the generation of reactive oxygen species [66], which easily oxidize lipid molecules [67].

It is possible that antioxidants are relatively depleted in WNS-affected bats because of sustained immune responses against *G. destructans* that may start before bats enter deep hibernation or may commence during interbout arousals. Increased frequency of arousals probably also leads to reductions in antioxidants as they neutralize the additional free radicals. These two mechanisms in combination could present a situation where the production and replacement of antioxidants cannot occur in sufficient amounts to balance the system. Moreover, during hibernation, protein synthesis is slowed [38], therefore potentially inhibiting the replenishment of certain antioxidants. Since WNS-affected bats exhibit limited fat reserves [1], [5], [6], [7], these animals probably have diminished ability to produce additional proteins compared with unaffected bats. One additional possibility is that antioxidants are being shunted from the periphery to susceptible tissues in WNS-affected bats to protect these tissues against oxidation [68]; however, this would likely be a temporary response that may not be protective throughout hibernation.

Because we did not concurrently measure products of lipid peroxidation, such as malonaldehyde, it is difficult to determine if little brown myotis hibernating in WNS-affected sites are indeed experiencing oxidative stress. These measures should be made to assess the relative degree of oxidative stress that affected bats may be experiencing; and the ability of phagocytes to engulf *G. destructans* and produce intracellular and extracellular reactive oxygen and nitrogen species should also be tested.

### Circulating Cytokines

We observed consistent results in only one of the analytes detected (IL-4) and it is difficult to tell if negative results associated with other cytokines of interest are due to a lack of cross-reactivity between mouse-specific reagents and bat cytokines or an absence of specific cytokines in circulation. Nevertheless, our results show that bats hibernating in WNS-affected sites have significantly lower IL-4 levels compared with bats from unaffected sites. Additionally, within affected sites only, visibly symptomatic bats showed lower IL-4 levels than asymptomatic bats. There may be a polarization of T lymphocyte responses in favor of T_H_1 responses in infected bats given that inhibition of IL-4 is known to decrease the activation of the T_H_2 subset [69]. Additionally, if visible signs of fungal infections are any indication of infection status, visibly symptomatic bats may be in a later stage of infection and immune activation. It is possible that infected individuals are attempting resistance through T cell mediated responses, which would result in activation of phagocytes at the site of infection. However, without the measurement of additional cytokines, particularly IFN-γ, it is difficult to know if the appropriate stimuli for activation of the T_H_1 subset are present. This could be an extremely important avenue of further inquiry because T_H_1 responses promote effective fungal clearance (i.e. memory responses) and increase inflammation in other taxa, whereas T_H_2 responses are inhibitory to the control of fungal infections [34].

### Conclusions

While the aspects of immune response measured in this study are an important selection of responses that might occur against *G. destructans*, it is evident that these processes respond to this disease to different degrees and even in different directions. Underlying mechanisms and the biological meaning of the observed changes are yet to be described. Thus, future studies should identify and test responses specifically against *G. destructans* (e.g. measure anti-*G. destructans* antibodies) in addition to the measurement of general immune parameters (e.g. total circulating leukocytes). Additionally, controlled captive studies that measure immune responses combined with measures of infection (i.e. histology) over a time course could provide clearer evidence of what capability little brown myotis have for successful immunological resistance against *G. destructans*. Regardless, this study demonstrates that hibernating little brown myotis are capable of certain immunological responses to WNS and suggests that investigating the role of oxidative tissue damage in WNS pathogenesis is warranted.

## Supporting Information

Table S1 **Statistics for all outcome variables used in this study, including total circulating leukocytes, total circulating immunoglobulins, total antioxidant power, and IL-4.**
(XLSX)
