# Correction: Hibernating Little Brown Myotis (*Myotis lucifugus*) Show Variable Immunological Responses to White-Nose Syndrome

**DOI:** 10.1371/annotation/2296467f-c10a-4677-8520-490a614ce355

**Published:** 2013-10-04

**Authors:** Marianne S. Moore, Jonathan D. Reichard, Timothy D. Murtha, Morgan L. Nabhan, Rachel E. Pian, Jennifer S. Ferreira, Thomas H. Kunz

The last sentence of the section "Total Circulating Leukocytes" should have cited reference 8 instead of 51.

The correct sentence should read: However, bats typically rewarm to euthermia more rapidly than other hibernators and may have arousal bouts under 90 minutes [8] making it difficult to predict changes in circulating leukocytes given the extent to which they differ from other taxa. 

